# Preoperative forced expiratory volume in one second and postoperative respiratory outcomes in nonpulmonary and noncardiac surgery: a retrospective cohort study

**DOI:** 10.1186/s40981-024-00729-w

**Published:** 2024-07-25

**Authors:** Toshiyuki Mizota, Miho Hamada, Akiko Hirotsu, Li Dong, Shino Matsukawa, Chikashi Takeda, Moritoki Egi

**Affiliations:** Department of Anesthesia, Kyoto University Hospital, 54 Shogoin-Kawahara-Cho, Sakyo-Ku, Kyoto, 606-8507 Japan

**Keywords:** Pulmonary function test, Forced expiratory volumes, Respiratory failure, Postoperative complications

## Abstract

**Background:**

Although the usefulness of pulmonary function tests has been established for lung resection and coronary artery bypass surgeries, the association between preoperative pulmonary function test and postoperative respiratory complications in nonpulmonary and noncardiac surgery is inconclusive. The purpose of this study was to determine the association between preoperative forced expiratory volume in one second (FEV1) on pulmonary function test and the development of postoperative respiratory failure and/or death in patients undergoing major nonpulmonary and noncardiac surgery.

**Methods:**

Adult patients aged ≥ 18 years and who underwent nonpulmonary and noncardiac surgery with expected moderate to high risk of perioperative complications from June 2012 to March 2019 were included. The primary exposure was preoperative FEV1 measured by pulmonary function test within six months before surgery. The primary outcome was respiratory failure (i.e., invasive positive pressure ventilation for at least 24 h after surgery or reintubation) and/or death within 30 days after surgery. A logistic regression model was used to adjust for the respiratory failure risk index, which is a scoring system that predicts the probability of postoperative respiratory failure based on patient and surgical factors, and to examine the association between preoperative FEV1 and the development of postoperative respiratory failure and/or death.

**Results:**

Respiratory failure and/or death occurred within 30 days after surgery in 52 (0.9%) of 5562 participants. The incidence of respiratory failure and/or death in patients with *FEV1* ≥ 80%, 70%– < 80%, 60%– < 70%, and < 60% was 0.9%, 0.6%, 1.7%, and 1.2%, respectively. Multivariable logistic regression analysis showed no significant association between preoperative FEV1 and postoperative respiratory failure and/or death (adjusted odds ratio per 10% decrease in FEV1: 1.01, 95% confidence interval: 0.88–1.17, *P* = 0.838). Addition of FEV1 information to the respiratory failure risk index did not improve the prediction of respiratory failure and/or death [area under the receiver operating characteristics curve: 0.78 (95% confidence interval: 0.72–0.84) and 0.78 (95% confidence interval: 0.72–0.84), respectively; *P* = 0.84].

**Conclusion:**

We found no association between preoperative FEV1 and postoperative respiratory failure and/or death in patients undergoing major nonpulmonary and noncardiac surgery.

**Supplementary Information:**

The online version contains supplementary material available at 10.1186/s40981-024-00729-w.

## Background

Respiratory complications are one of the most important perioperative complications that can significantly affect the recovery of patients who undergo surgery. Among them, respiratory failure is the most severe form and has been shown to be associated with a significant increase inhospital mortality [1, 2]. Prediction of the risk for postoperative respiratory failure is important in patient management, including the decision on the choice of treatment and resource utilization, such as the intensive care unit.

Consensus on the value of preoperative pulmonary function tests (PFTs) is available for lung resection surgery and coronary artery bypass graft surgery [3]. On the other hand, the value of PFTs in nonpulmonary and noncardiac surgery is inconclusive. Clinical guidelines developed by the American College of Physicians in 2006 [4] state that preoperative PFT should not be used routinely to predict the risk of postoperative pulmonary complications because PFT data are not consistently superior to history or physical examination in predicting postoperative pulmonary complications. However, in a systematic review conducted to develop these guidelines [3], only four small studies examined the independent association between PFT data and postoperative pulmonary complications, which were insufficient to conclude an association between PFT data and postoperative pulmonary complications. Subsequently published studies that evaluated the association between preoperative PFTs and postoperative respiratory complications after adjusting for clinically relevant variables have shown inconsistent results [5–9]. The reasons for these inconsistent results are not clear but may include differences in sample size, type of surgery, or the definition of respiratory complications. In addition, none of these studies examined the association between preoperative PFT results and postoperative respiratory failure, which is the most severe form of respiratory complication.

The respiratory failure risk index (RFRI) is a simple scoring system that predicts the probability of postoperative respiratory failure based on five patient factors, including age, history of chronic obstructive pulmonary disease, disability, preoperative blood urea nitrogen level, and preoperative serum albumin level, and two surgical factors, including type of surgery and emergency surgery [10]. However, the RFRI has not been validated with external data. Furthermore, no studies have evaluated the predictive value of adding the PFT results to the known respiratory complication scoring systems, such as the RFRI.

In this study, we investigated the association between the forced expiratory volume in one second (FEV1) measured by PFT and the development of postoperative respiratory failure and/or death. We tested the hypothesis that a decrease in the FEV1 is associated with the development of postoperative respiratory failure and has additional value to the RFRI in predicting postoperative respiratory failure.

## Methods

### Study design, setting, and population

This single-center retrospective cohort study was conducted at Kyoto University Hospital, which is a 1121-bed teaching hospital. The ethics committee of the Kyoto University Graduate School of Medicine approved the study protocol (approval number: R2646, September 17, 2020) and waived the requirement for informed consent because of retrospective nature of this study. This study adhered to the Strengthening the Reporting of Observational Studies in Epidemiology statement [11]. Patients aged ≥ 18 years and who underwent major nonpulmonary and noncardiac surgery under general anesthesia at Kyoto University Hospital from June 2012 to March 2019, which was the duration of the availability of PFT data, were included. We defined major nonpulmonary and noncardiac surgery as those procedures classified as intermediate or high-risk surgery according to the 2014 European Society of Cardiology/European Society of Anaesthesiology guidelines, excluding cardiac and pulmonary surgery [12]. The specific procedures of major nonpulmonary and noncardiac surgery were as follows: abdominal aortic aneurysm repair, carotid endarterectomy, esophagectomy, gastrectomy, colorectal resection, liver resection, biliary tract surgery, pancreatic resection, splenectomy, nephrectomy, adrenalectomy, cystectomy, total pelvic organ removal, total hip arthroplasty, total knee arthroplasty, lower extremity amputation, peripheral vascular bypass surgery, and spine surgery. Patients who underwent organ transplant surgery or emergency surgery, those who were on preoperative respiratory support, and those who had not undergone a PFT within 6 months prior to surgery were excluded.

### Data collection

Data were collected from the Kyoto University Hospital IMProve Anesthesia Care and ouTcomes database [13], which aimed to clarify the relationship of intraoperative respiratory and cardiovascular parameters with postoperative outcomes. In addition, data from PFTs performed 6 months before surgery were collected. The definitions of the collected variables are shown in Supplemental Table 1.

**Table 1:** Baseline and operative characteristics and outcomes of 5562 participants

Characteristics	*N* = 5562
Age	
18–59 years	1483 (26.7%)
60–69 years	1553 (27.9%)
≥ 70 years	2526 (45.4%)
Sex	
Female	2730 (49.1%)
Male	2832 (50.9%)
Body mass index (kg/m^2^)	22.7 (20.6–25.3)
ASA-PS	
I	915 (16.5%)
II	4144 (74.7%)
III	487 (8.8%)
IV	1 (0.0%)
Partially or fully dependent status	633 (11.4%)
Chronic obstructive pulmonary disease	109 (2.0%)
Congestive heart failure	522 (9.4%)
Preoperative hemoglobin (g/dL)	12.7 (11.4–13.8)
Preoperative serum albumin (g/dL)	3.9 (3.6–4.2)
Preoperative blood urea nitrogen (mg/dL)	15 (12–19)
Respiratory failure risk index	14 (6–20)
PFT results	
FEV1 (% predicted)	95.9 (83.6–107.3)
FVC (% predicted)	100.6 (89.7–111.1)
VC (% predicted)	100.1 (89.5–110.1)
Type of surgery	
Upper abdominal surgery	2824 (50.8%)
Lower abdominal surgery	562 (10.1%)
Thoracic surgery	167 (3.0%)
Abdominal aortic aneurysm	108 (1.9%)
Peripheral vascular surgery	41 (0.7%)
Extremities	1291 (23.2%)
Spine surgery	569 (10.2%)
Operation time (min)	265 (146–409)
Blood loss (mL)	80 (0–320)
Postoperative respiratory failure and/or death	52 (0.9%)
Postoperative respiratory failure within 30 days of surgery	44 (0.8%)
30-day mortality	15 (0.3%)
Inhospital mortality	22 (0.4%)
Postoperative hospital length of stay (days)	18 (13–25)

*FEV1* forced expiratory volume in one second, *FVC* forced vital capacity, *PFT* pulmonary function test, *VC* vital capacity

### Exposures

The primary exposure was preoperative FEV1 within 6 months before surgery. If more than one PFT was performed within 6 months before surgery, the most recent value was used. In addition, the forced vital capacity (FVC) and the vital capacity (VC) were considered as exploratory exposure factors. FEV1, FVC, and VC were used in the analysis by determining the % predicted values, which were calculated based on age, sex, and height and have been validated in the Japanese population [14]. In the logistic regression analyses, the FEV1% predicted and FVC % predicted were reverse-coded so that the lower values were associated with higher hazard ratios.

### Outcomes

The primary outcome was respiratory failure and/or death within 30 days after surgery. Respiratory failure was defined as mechanical ventilation by endotracheal tube or tracheostomy for more than 24 h after surgery or postoperative reintubation. The secondary outcomes were 30-day mortality, inhospital mortality, and postoperative hospital length of stay. Postoperative length of stay was recorded for patients who survived until discharge.

### Statistical analyses

First, we performed an external validation of the RFRI. As reported in a previous study [10], the RFRI was incorporated into a logistic regression model after the following transformation:

*ln* (RFRI + 1),

where *ln* indicated the natural logarithm. We graphically assessed the calibration of the RFRI with a calibration plot and tested it with the Hosmer–Lemeshow test. A *P*-value of < 0.05 indicated a lack of good fit for the model. For model discrimination, we computed the area under the receiver operating characteristic curve (AUROC) with a 95% confidence interval (CI) using 500 bootstrap resampling.

Next, we performed a logistic regression analysis after adding FEV1 as a continuous variable to the RFRI to determine whether FEV1 was associated with respiratory failure and/or death independent of the RFRI. Because the association between preoperative FEV1 and the development of postoperative respiratory failure may not be linear, we used a restricted cubic spline of five knots and a categorized FEV1 into four based on Goldman’s classification (i.e., normal: ≥ 80%, mild decline: 70% to < 80%, moderate decline: 60% to < 70%, and severe decline: < 60%) to evaluate the existence of nonlinear relationships. In addition, we assessed the impact of adding the value of FEV1 to the RFRI in predicting respiratory failure and/or death by comparing AUROC values.

Furthermore, we examined the associations of FVC and VC with respiratory failure and/or death by adjusting for the RFRI on logistic regression analysis. In this analysis, the FVC and VC were entered into the model as continuous variables, and the odds ratios (ORs) per 10% decline were determined. According to the American College of Physicians guidelines, preoperative spirometry is recommended only in high-risk patients [4]. Therefore, according to the median RFRI, we stratified the patients into high-risk (*RFRI* ≥ 15) and low-risk (*RFRI* ≤ 14) groups and evaluated the association between FEV1 and respiratory failure and/or death in each group. In addition, we performed a subgroup analysis of abdominal and nonabdominal surgery groups, because decreased preoperative pulmonary function was reported to be associated with the occurrence of respiratory complications in abdominal surgery [7, 8].

We assessed the robustness of our findings using two sensitivity analyses. Sensitivity models were constructed as a logistic regression that was identical with the primary model described above, except for the following: (i) outcome was redefined as respiratory failure and/or death within 7 days instead of 30 days after surgery and (ii) with adjustments for factors that were reported to be risk factors for postoperative respiratory complications but were not included in the RFRI (i.e., congestive heart failure, preoperative hemoglobin level, and operative time) [4, 15].

We decided to use data from all cases included in the database to maximize the statistical power. For sample size estimation, 10 events per variable were required for reliable multivariable logistic regression analysis [16]. In our previous study on patients undergoing major abdominal surgery, 1.8% of patients received invasive respiratory support [17], and the estimated number of major nonpulmonary and noncardiac surgeries performed annually was 700. Based on these data, 4900 surgeries were calculated to meet the inclusion criteria, and 88 cases were calculated to develop the primary outcome of postoperative respiratory failure and/or death during the study period of approximately seven years. Therefore, the estimate was to perform a multivariable logistic regression with eight variables.

For missing data, the plan was to perform a complete case analysis if the percentage of missing data was < 5%; such an analysis was considered to be feasible [18]. If the percentage of missing data was > 5%, we planned to complete the missing values.

## Results

### Patient and operative characteristics and outcomes

**Fig. 1:**
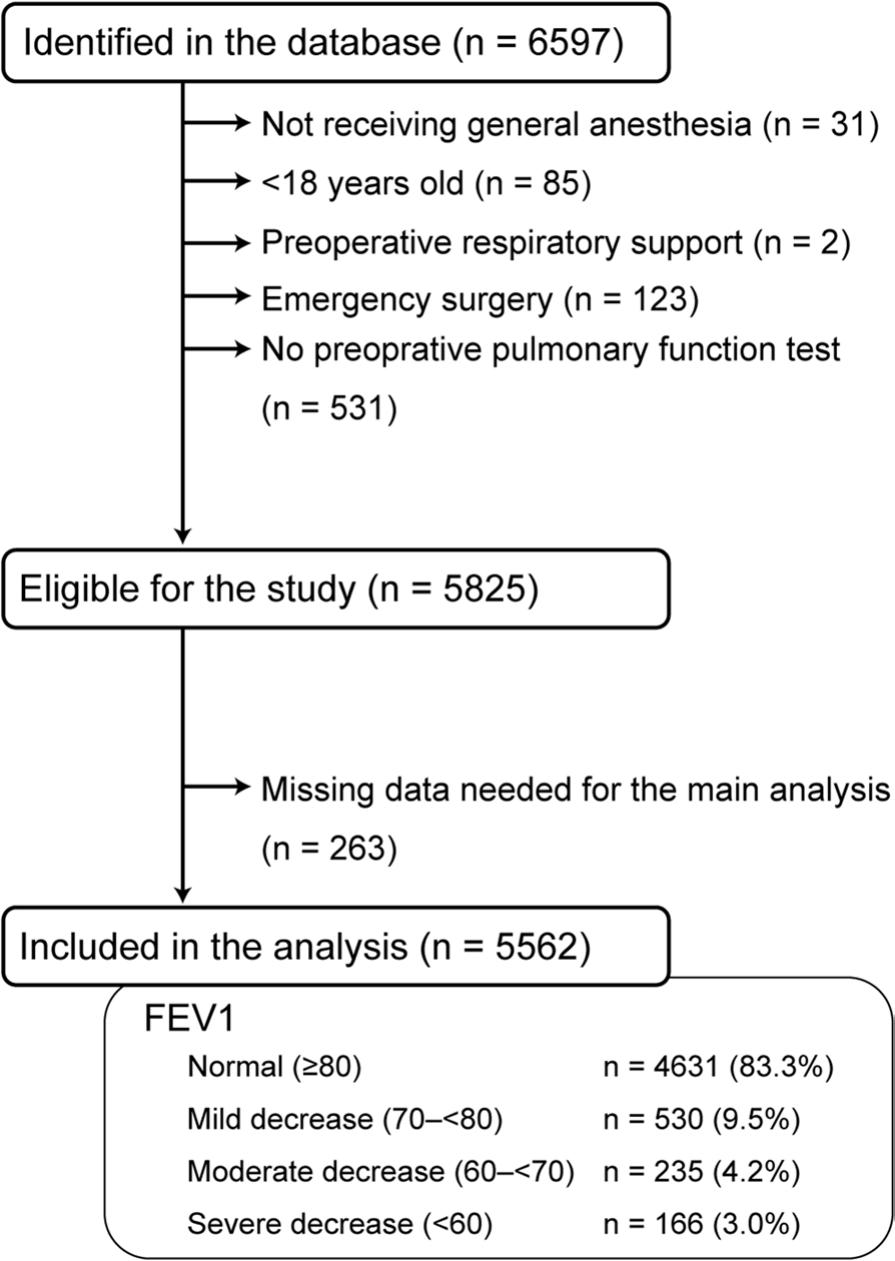
Flow diagram of study participants

The flow diagram of the study is shown in Fig. 1. Of the 6597 patients identified in the database to have undergone major nonpulmonary and noncardiac surgery, 5825 met the inclusion and exclusion criteria. Of these, 263 patients (4.5%) had missing data on the variables required to calculate the RFRI. Because < 5% of the patients had missing data, we decided to perform a complete case analysis, which resulted in 5562 patients being included in the analysis. Of the 5562 patients in total, 4631 (83.3%) had FEV1 values within the normal range (≥ 80%), and 9.5%, 4.2%, and 3.0% had mild, moderate, and severe FEV1 decline, respectively. Table 1 shows the patient and operative characteristics and outcomes. Respiratory failure and/or death within 30 days after surgery occurred in 52 patients (0.9%); 44 developed respiratory failure, and 15 died within 30 days of surgery.

### External validation of the RFRI

**Fig. 2:**
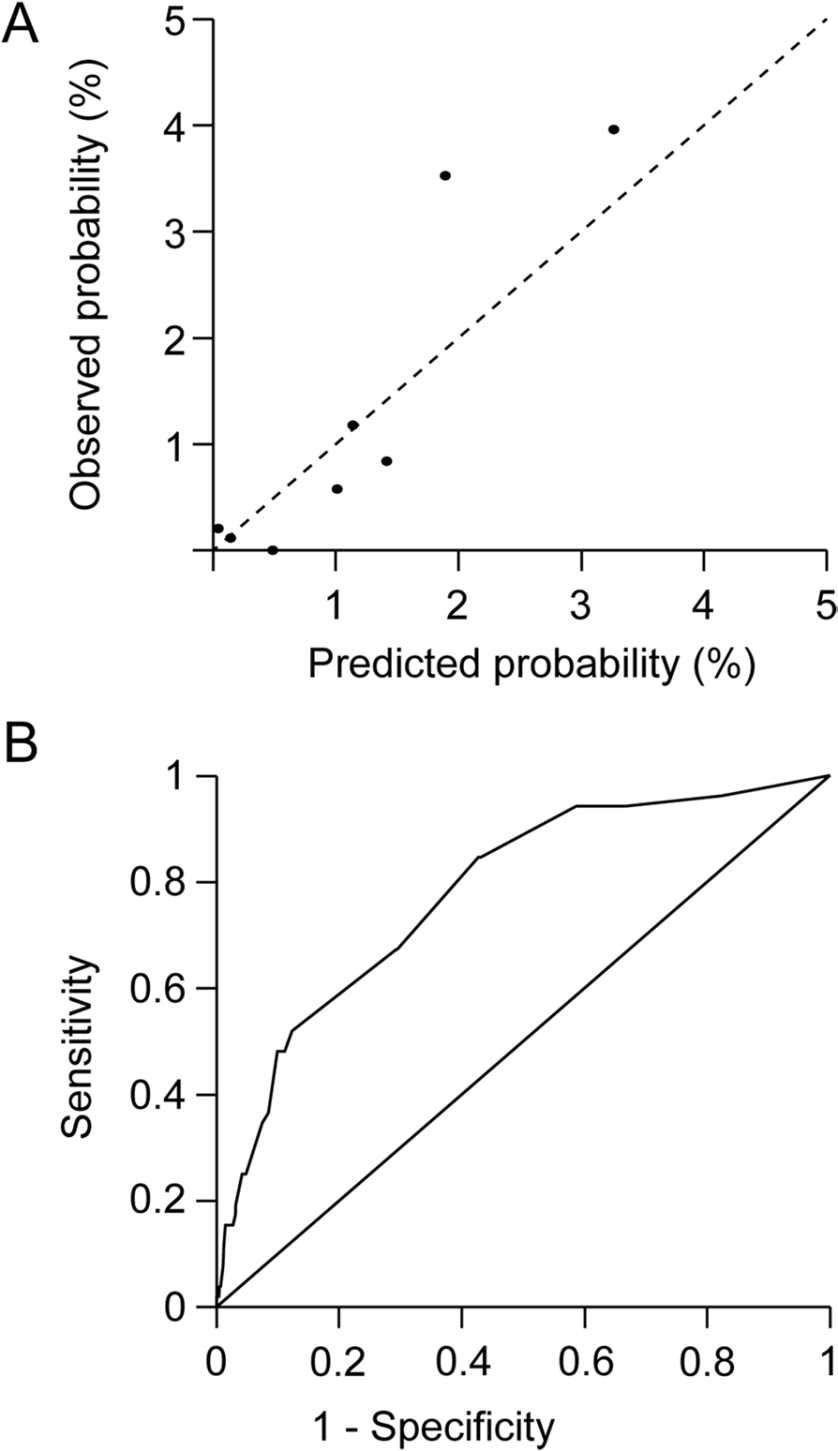
**A** The calibration plot of RFRI for predicting the probability of respiratory failure and/or death. **B** The receiver operating characteristic curve for prediction of respiratory failure and/or death based on RFRI

We first evaluated the performance of RFRI in predicting respiratory failure and/or death in terms of calibration and discrimination. RFRI tended to underestimate the probability of respiratory failure and/or death in the group of patients with high predicted probability (*P* for Hosmer–Lemeshow test: 0.010; Fig. 2A). The AUROC for the RFRI was 0.78 (95% *CI*: 0.72–0.84; Fig. 2B).

### Evaluation of FEV1 as a predictor of respiratory failure and/or death

In the analysis that treated FEV1 as a continuous variable, the unadjusted OR for the development of respiratory failure and/or death was 1.12 per 10% decrease in FEV1 (95% *CI*: 0.97–1.29, *P* = 0.133), and the adjusted OR for the RFRI was 1.01 per 10% decrease in FEV1 (95% *CI*: 0.88–1.17, *P* = 0.838). In the multivariable logistic model, the restricted cubic spline of the FEV1 showed that its nonlinear effect on respiratory failure and/or death was not significant (all nonlinear effect *P* > 0.05 with knots = 3, 4, and 5 in spline function); therefore, there was no need to include a cubic term in the model.

**Fig. 3:**
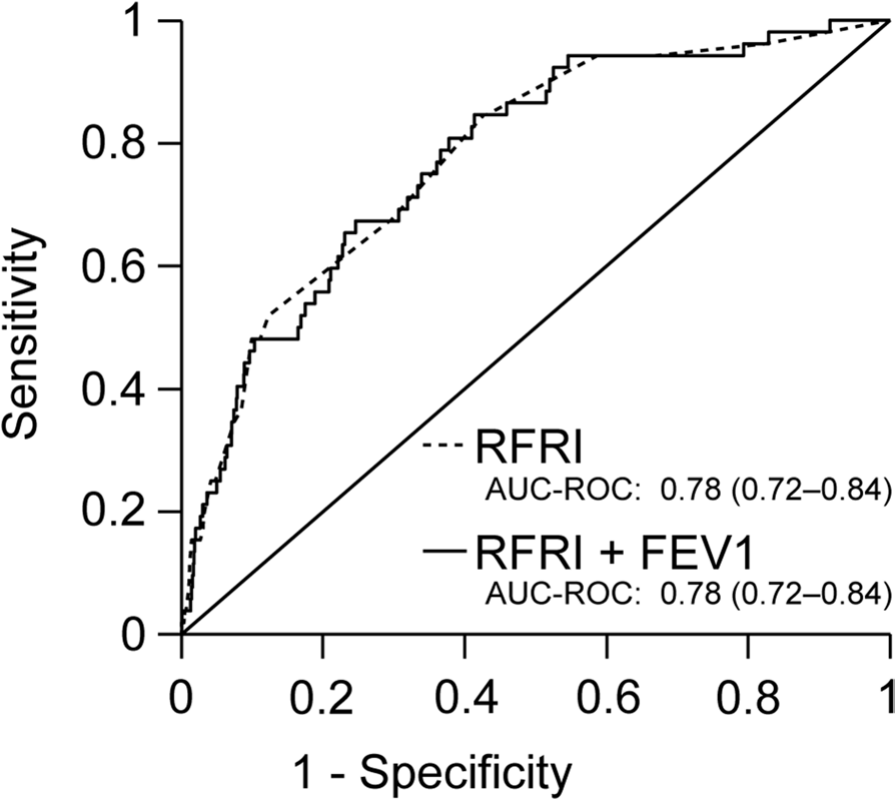
The area under the receiver operating characteristic curves for respiratory failure and/or death as predicted by RFRI alone and FEV1 combined with RFRI. RFRI, respiratory failure risk index; FEV1, forced expiratory volume in one second

In the analysis that treated FEV1 as a categorical variable, FEV1 70% to < 80%, 60% to < 70%, and < 60% were not associated with a significant increase in respiratory failure and/or death compared with *FEV1* ≥ 80% (Table 2). The addition of FEV1 as a continuous variable to the prediction of respiratory failure and/or death by RFRI did not significantly increase the AUC (Fig. 3).

**Table 2:** Observed outcomes and adjusted risk of developing respiratory failure and/or death by four categories of FEV1

FEV1 (% predicted)	No. of patients with respiratory failure and/or death/total no	Adjusted odds ratio (95% confidence interval)	*p*-value
≥ 80%	43/4631	-	-
70%– < 80%	3/530	0.46 (0.14–1.48)	0.192
60%– < 70%	4/235	1.22 (0.43–3.48)	0.712
< 60%	2/166	0.71 (1.67–2.99)	0.636

*FEV1* forced expiratory volume in one second

Both FVC and VC were not associated with respiratory failure and/or death. The adjusted OR per 10% decrease in FVC was 1.08 (95% *CI*: 0.93–1.27, *P* = 0.317), and the adjusted OR per 10% decrease in VC was 1.07 (95% *CI*: 0.90–1.27, *P* = 0.437).

### Relationship between FEV1 and secondary outcomes

Secondary outcomes stratified by four categories of FEV1 are presented in Table 3. FEV1 was not significantly associated with 30-day mortality (adjusted *OR*: 0.85 per 10% decrease; 95% *CI*: 0.65–1.10; *P* = 0.219), inhospital mortality (adjusted *OR*: 1.07 per 10% decrease; 95% *CI*: 0.86–1.32; *P* = 0.545), or postoperative hospital length of stay (*β* coefficient: 0.19 per 10% decrease; 95% *CI*: − 0.07–0.45; *P* = 0.157).

**Table 3:** Secondary outcomes by four categories of FEV1

FEV1 (% predicted)	No. of patients who died within 30 days after surgery/total no	No. of patients who died during postoperative hospitalization/total no	Postoperative days in hospital (mean ± standard deviation)
≥ 80%	14/4631	15/4631	22.0 ± 18.2
70%– < 80%	0/530	5/530	23.4 ± 19.5
60%– < 70%	1/235	1/235	24.0 ± 20.2
< 60%	0/166	1/166	24.6 ± 17.4

*FEV1* forced expiratory volume in one second

### Subgroup and sensitivity analyses

**Fig. 4:**
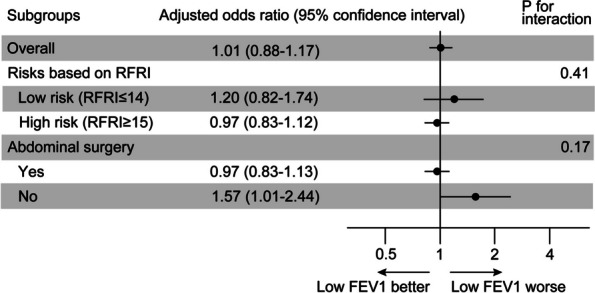
Subgroup analyses stratified by respiratory failure risk and operation site

Subgroup analysis based on the RFRI and surgical site suggested that there was no interaction between these variables and FEV1, although there was a weak association between lower FEV1 and postoperative respiratory failure and/or death in nonabdominal surgery (Fig. 4). The relationship between preoperative FEV1 and postoperative respiratory failure and/or death remained qualitatively unchanged in the sensitivity analyses (Table 4).

**Table 4:** Sensitivity analyses for the association between preoperative FEV1 and postoperative respiratory failure and/or death

	Adjusted odds ratio (95% confidence interval)	*p*-value
Restricting assessment window for respiratory failure and/ or death to seven PODs	0.92 (0.77–1.11)	0.400
Adjusting for congestive heart failure, preoperative hemoglobin level, and operative time	1.01 (0.86–1.18)	0.947

*FEV1* forced expiratory volume in one second, *POD* postoperative day

## Discussion

In this study, we externally validated the RFRI in predicting postoperative respiratory failure and/or death and found acceptable discrimination, although it tended to underestimate the risk in the group of patients with high predictive probability. The preoperative FEV1 measured by PFT was not significantly associated with the development of postoperative respiratory failure and/or death, and the addition of FEV1 to RFRI did not improve the prediction of respiratory failure and/or death. Even a severe decline in FEV1 (i.e., < 60% predicted) was not significantly associated with postoperative respiratory failure and/or death. These results suggested that preoperative PFT is of little use for risk stratification of postoperative respiratory failure in patients undergoing nonpulmonary and noncardiac surgery.

Although there was a study that showed the utility of the RFRI in predicting postoperative respiratory complications, including respiratory failure and pneumonia [19], to our knowledge, there had been no study that externally validated the ability of the RFRI to predict postoperative respiratory failure. In this present study, we showed that the RFRI, which can be calculated from readily available variables, had acceptable discrimination and was useful for risk stratification of patients undergoing nonpulmonary and noncardiac surgery. However, the RFRI may underestimate the probability of respiratory failure and/or death in a group of patients with high predicted probability, and improving the predictive performance of the RFRI in this group is a future challenge.

The results of this study, which found no significant association between preoperative FEV1 and postoperative respiratory failure and/or death, were inconsistent with the results of several previous studies. Tajima et al. [7] reported that preoperative VC was predictive of postoperative pneumonia reported in 1236 patients who underwent colorectal cancer surgery. Oh et al. [8] found that low preoperative FVC was associated with postoperative respiratory complications in 898 high-risk patients who underwent laparoscopic abdominal surgery. Jeong et al. [9] reported that airflow limitation, defined as a decrease in FEV1, was associated with postoperative pulmonary complications in 2059 patients who underwent non-pulmonary/non-obstetric surgery. While patient characteristics, such as age and body mass index, were similar between this study and previous studies [7–9], there were some differences. The first was the surgical site. The two previous studies were conducted on patients who underwent abdominal surgery, whereas this study included patients who underwent a wide range of surgeries, including spine and extremity surgeries. Although the association between pulmonary function and postoperative respiratory complications may vary according to the surgical site, the present study failed to show an association between preoperative FEV1 and respiratory failure and/or death in both the entire population and in the subgroup of abdominal surgery. The next difference is the primary exposure factor; the two previous studies used VC or FVC, whereas this study used FEV1. However, we also performed a subanalysis using VC and FVC as exposure factors in this study and found no association between pulmonary function and respiratory failure and/or death. Furthermore, the primary outcome differed between this study and the previous studies; the two previous studies used respiratory complications, such as atelectasis, pneumonia, and pulmonary edema, whereas this study used respiratory failure and/or death. Further verification is needed to clarify which of these differences produced the difference in results between this study and the previous studies.

This study had several strengths. First, the availability of preoperative PFTs in > 90% of the patients made this study suitable for the assessment of the predictive value of routine preoperative PFTs in major surgery. In previous studies, PFTs may have been performed only in patients who were judged to be at high risk based on clinical findings; this design would not be suitable for evaluating the usefulness of routine preoperative PFTs. To our best knowledge, this study was the first to evaluate the ability of PFTs in predicting respiratory failure and/or death, which is the most serious respiratory complication and a clinically relevant outcome.

This study had several limitations that should be considered when interpreting the results. Being a single-center study, generalizability is limited; external validation is needed to corroborate our findings. Although the present study had the largest sample size among the studies that examined the association between preoperative PFT and postoperative respiratory complications, the occurrence of respiratory failure was infrequent. Therefore, even the large sample size of this study may have had insufficient power. However, the adjusted OR for respiratory failure and/or death was almost equal to 1.0 for every 10% decrease in FEV1, and there were no differences in postoperative length of hospital stay and survival outcome among the FEV1 categories, suggesting that decreased preoperative FEV1 did not have a fundamental impact on the postoperative course. The definition of respiratory failure varies across studies, and different results may be obtained depending on the setting of the outcome. Because this was a retrospective study, the perioperative management strategies were not standardized. Therefore, we cannot exclude the possibility that the association between preoperative FEV1 and postoperative respiratory failure and/or death may have been counteracted by more intensive treatment and rehabilitation in patients with low FEV1.

## Conclusions

We found no association between preoperative FEV1 and postoperative respiratory failure and/or death in patients who underwent nonpulmonary and noncardiac surgery.

### Supplementary Information

Supplemental Table 1. Definitions of the collected variables.

## Data Availability

The datasets used and/or analyzed during the current study are available from the corresponding author on reasonable request.

## Abbreviations

FEV1: Forced expiratory volume in one second
PFT: Pulmonary function test
RFRI: Respiratory failure risk index
FVC: Forced vital capacity
VC: Vital capacity
AUROC: Area under the receiver operating characteristic curve
CI: Confidence interval
OR: Odds ratio
