# Supplementary material for: Preoperative forced expiratory volume in one second and postoperative respiratory outcomes in nonpulmonary and noncardiac surgery: a retrospective cohort study
Source: JA Clin Rep. 2024 Jul 25;10:44. doi: 10.1186/s40981-024-00729-w (PMC11272759; doi:10.1186/s40981-024-00729-w)
Supplement: Supplementary file 1 — Supplemental Table 1. Definitions of the collected variables. [file 40981_2024_729_MOESM1_ESM.docx]

**Supplemental Material**

**Supplemental Table 1.** Definitions of the collected variables

| Variables | Definitions |
| --- | --- |
| Age | In years |
| Sex | Male or female |
| Height, weight, and body mass index | Most recent value measured before the surgery |
| ASA-PS | 1 to 5, recorded in the electronic database |
| Partially or fully dependent status | Needs assistance from another person for any of the following items in the assessment using the Katz index of independence in activities of daily living at the time of admission: bathing, dressing, toileting, transferring, continence, and feeding |
| Chronic obstructive pulmonary disease | ICD-10 code J41–J44 and preoperative FEV1 <70% |
| Congestive heart failure | ICD-10 code I099, I110, I130, I132, I255, I420, I425–I429, I43, I50, P290 |
| Preoperative hemoglobin | In g/dL, most recent value measured before surgery |
| Preoperative albumin | In g/dL, most recent value measured before surgery |
| Preoperative blood urea nitrogen | In mg/dL, most recent value measured before surgery |
| RFRI | Obtained by adding the points assigned to each of the five patient factors and two surgical factors (Ann Surg 2000; 232: 242–253) |
| Duration of surgery | In minutes, from skin incision to skin closure |
| Intraoperative blood loss | In mL, extracted from the electronic database |
| Postoperative respiratory failure and/or death | Mechanical ventilation by endotracheal tube or tracheostomy for more than 24 hours after surgery or postoperative reintubation |
| 30-day mortality | Death within 30 days after surgery |
| In-hospital mortality | Death during the hospital stay for the surgery |
| Postoperative hospital length of stay | Number of days from surgery to discharge from the hospital |

*ASA-PS, American Society of Anaesthesiologists physical status; ICD-10, International Classification of Diseases tenth revision; FEV1, forced expiratory volume in one second; RFRI, Respiratory Failure Risk Index*
